# Recent insights into perceptual and motor skill learning

**DOI:** 10.3389/fnhum.2014.00683

**Published:** 2014-09-03

**Authors:** Lior Shmuelof, John W. Krakauer

**Affiliations:** ^1^Department of Brain and Cognitive Sciences, Ben-Gurion University of the NegevBeer-Sheva, Israel; ^2^Departments of Neurology and Neuroscience, Johns Hopkins UniversityBaltimore, MD, USA

**Keywords:** explicit learning, implicit learning, acuity, neural plasticity, intersubject variability, age differences

Improvements in task performance following practice can occur as a result of changes in distinct cognitive and neural processes. In some cases, we can improve our performance by selecting a more successful behavior that is already part of our available repertoire. Skill learning, on the other hand, refers to a slower process that results in improving the ability to perform a behavior, i.e., it involves the acquisition of a behavior that was not available to the controller before training. Skill learning can take place both in sensory and in motor tasks. Thus, sensory skill acquisition using perceptual learning tasks is measured by improvements in sensory acuity through practice-induced changes in the sensitivity of relevant neural networks (Poggio et al., 1992). Motor skill is harder to define as the term is used whenever a motor learning behavior improves along some dimension. We have recently argued that as in perceptual learning, acuity is an integral component in motor skill learning (Shmuelof et al., 2012). Surprisingly, comparisons between perceptual and motor learning are largely lacking in the literature (Censor et al., 2012). In this special topic we set out to integrate experimental and theoretical work on perceptual and motor skill learning and to stimulate a discussion regarding the similarities and differences between these two kinds of learning.

Stanley and Krakauer use the famous case of H.M. to argue that while motor acuity may not require intact explicit memory mechanisms (Milner, 1962), the process of acquisition of a new motor skill does require knowledge of facts in order to assemble a novel action (Stanley and Krakauer, 2013). This assertion also suggests a fundamental difference between motor and perceptual skills—while motor skills require intention and knowledge of facts, perceptual skills can be learned implicitly outside of conscious awareness. Series and Seitz review studies that examine the effect of experience on visual expectation using a Bayesian framework. Findings suggest that expectations are constructed based on structural and contextual features, and that with task experience, performance may be enhanced not only through increased ability to detect or to act, but also by biasing response selection according to the statistics of the environment (Series and Seitz, 2013). Focusing on the physiological basis of visual acuity, Rokem and Silver show that performance gains in a visual discrimination task induced by cholinergic enhancement during training, last between 5 and 15 months after training termination, which demonstrates that manipulating neural plasticity at an early stage of learning can have long-lasting effects on a perceptual ability (Rokem and Silver, 2013). Interestingly, the fact that a skill can be retained over the long term does not immunize it from potential modifications at later periods. Yotsumoto and colleagues show that sequence learning is susceptible to interference even after training on a finger-tapping task for 8 days (Yotsumoto et al., 2013).

Another aspect of skill learning that is often overlooked is the influence that the initial state of the system has on subsequent acquisition. Thus, the same training protocol can lead to different outcomes depending on the initial ability and state of the actor. Using the serial reaction time task, Nemeth and colleagues show that implicit sequence learning ability gradually decreases after the age of 12, whereas explicit learning of sequences is not affected by age, suggesting a dissociation of implicit and explicit learning processes throughout development (Nemeth et al., 2013). Furthermore, Mayhew and Kourtzi demonstrate that while young and older adults show comparable improvements in a visual discrimination task; these changes in performance are subserved by different neuronal networks (Mayhew and Kourtzi, 2013). The importance of the initial state on learning is also observed within the same age group. Kostrubiec and colleagues show that the intrinsic baseline dynamics of bimanual phase coordination affect the pattern of phase changes induced by training; subjects who exhibit a coordination patterns that is close to the trained pattern will shift their pattern, whereas subjects who don't have an approximate baseline pattern will generate a new pattern of phase coordination (Kostrubiec et al., 2012). Inter-individual differences in motor learning are also reported by Park and colleagues where they show that both task-relevant and task irrelevant performance variables in bimanual coordination tasks are retained even after 8 years (with no training) (Park et al., 2013). Taken together, these studies clearly indicate that skill learning depends on an interaction between subjects' initial performance and subsequent task requirements, and that comparable improvements in task performance may be a result of different underlying processes, depending on age and other sources of inter-subject variability.

Multiple experimental approaches can be taken to investigate the neural mechanisms of skill acquisition and retention. Shabbott and colleagues show that patients with Huntington's disease, a neurodegenerative basal ganglia disorder, suffer from impairments in motor skill learning even after accounting for their inferior baseline motor performance compared to control subjects (Shabbott et al., 2013). Orsborn and Carmena review how a brain machine interface can be used to perform closed-loop experiments to better understand the neural changes that underlie skill learning (Orsborn and Carmena, 2013). Acquisition of a skillful behavior is often composed of the sequential progression from goal-directed action to habit. Gremel and Costa focus on the premotor cortex of mice, and show that while mice can acquire new behaviors even after lesions to the premotor cortex (M2), these lesions affect subsequent desensitization to target reward, pointing to the M2's role in habit formation (Gremel and Costa, 2013).

Overall, while the term skill is frequently used to praise sports players and musicians, the scientific community still struggles to understand the fundamental processes underlying skill acquisition, or to even agree on what the term should encompass. The diverse studies included in this special topic indicate the involvement of multiple learning processes and brain structures in the complex process of skill acquisition. Critically, the level of skill achieved and its long-term retention depends both on the initial state of the learner and subsequent training conditions. Progress in this field will depend on identifying both the general theoretical rules of practice across perceptual and motor domains, and the unique empirical features of particular circuits and brain structures.

## Conflict of interest statement

The authors declare that the research was conducted in the absence of any commercial or financial relationships that could be construed as a potential conflict of interest.
